# Preoperative and postoperative prediction of long-term meningioma outcomes

**DOI:** 10.1371/journal.pone.0204161

**Published:** 2018-09-20

**Authors:** Efstathios D. Gennatas, Ashley Wu, Steve E. Braunstein, Olivier Morin, William C. Chen, Stephen T. Magill, Chetna Gopinath, Javier E. Villaneueva-Meyer, Arie Perry, Michael W. McDermott, Timothy D. Solberg, Gilmer Valdes, David R. Raleigh

**Affiliations:** 1 Department of Radiation Oncology, University of California San Francisco, San Francisco, United States of America; 2 Department of Neurological Surgery, University of California San Francisco, Sa Francisco, United States of America; 3 Department of Radiology, University of California San Francisco, San Francisco, United States of America; 4 Department of Pathology, University of California San Francisco, San Francisco, United States of America; California State University, UNITED STATES

## Abstract

**Background:**

Meningiomas are stratified according to tumor grade and extent of resection, often in isolation of other clinical variables. Here, we use machine learning (ML) to integrate demographic, clinical, radiographic and pathologic data to develop predictive models for meningioma outcomes.

**Methods and findings:**

We developed a comprehensive database containing information from 235 patients who underwent surgery for 257 meningiomas at a single institution from 1990 to 2015. The median follow-up was 4.3 years, and resection specimens were re-evaluated according to current diagnostic criteria, revealing 128 WHO grade I, 104 grade II and 25 grade III meningiomas. A series of ML algorithms were trained and tuned by nested resampling to create models based on preoperative features, conventional postoperative features, or both. We compared different algorithms’ accuracy as well as the unique insights they offered into the data. Machine learning models restricted to preoperative information, such as patient demographics and radiographic features, had similar accuracy for predicting local failure (AUC = 0.74) or overall survival (AUC = 0.68) as models based on meningioma grade and extent of resection (AUC = 0.73 and AUC = 0.72, respectively). Integrated models incorporating all available demographic, clinical, radiographic and pathologic data provided the most accurate estimates (AUC = 0.78 and AUC = 0.74, respectively). From these models, we developed decision trees and nomograms to estimate the risks of local failure or overall survival for meningioma patients.

**Conclusions:**

Clinical information has been historically underutilized in the prediction of meningioma outcomes. Predictive models trained on preoperative clinical data perform comparably to conventional models trained on meningioma grade and extent of resection. Combination of all available information can help stratify meningioma patients more accurately.

## Introduction

Meningioma is the most common primary cancer of the central nervous system, accounting for more than 30% of all brain tumors and more than 50% of benign intracranial neoplasms [1]. It is estimated that more than 25,000 meningiomas are diagnosed in the United States each year, and the majority are effectively managed with surgery, radiation, or a combination of both [2]. The World Health Organization (WHO) categorizes meningiomas into three grades based on mitotic activity and histopathologic characteristics [3]. Most WHO grade I meningiomas can be cured with gross total resection or definitive radiotherapy, but grade II (atypical) and grade III (anaplastic) meningiomas are prone to local recurrence and generally require adjuvant treatment [2]. There are no effective systemic agents for meningioma, and thus, patients with high grade or subtotally-resected meningiomas undergo serial craniotomy, radiotherapy and radiosurgery for recurrent disease, often leading to significant morbidity and even treatment-associated mortality [4,5].

Evolving understanding of the molecular genetics of meningiomas suggests that targeted agents may eventually improve treatments and outcomes for meningioma patients [6,7]. In the interim, meningioma patients are stratified and assigned to adjuvant treatment primarily according to tumor grade and extent of resection. Indeed, the majority of clinical data from meningioma patients, such as demographic and radiologic features, are largely ignored when prognosticating outcome. These data are omitted from clinical decisions because prospective, multi-institution trials have yet to identify clear features that influence meningioma outcomes [8,9], and no tractable algorithms or predictive models have been developed.

Machine learning (ML) enables the development of robust predictive models by identifying multivariate patterns in patient data that are related to clinical outcomes of interest. ML algorithms can incorporate a large number of variables of different data types (continuous, categorical or ordinal) in a single model, maximizing performance and minimizing problems associated with multiple comparisons. Unlike statistical hypothesis testing, machine learning focuses on prediction accuracy and offers ways to estimate model generalizability on unseen and future datasets, both of which are of critical importance in clinical practice. Aside from outcome prediction, different machine learning algorithms offer complementary ways of exploring and visualizing patterns in clinical data, which may provide new insights in disease pathophysiology and treatment. For meningioma, the random forest algorithm has been used to predict tumor grade from radiomic data [10]. Yet random forest is only one of a multitude of tractable ML algorithms, and according to the “No Free-Lunch Theorem,” no individual algorithm is guaranteed to perform best across all clinical scenarios [11]. Other algorithms could offer better performance in biomedical data analyses in terms of accuracy or interpretability, meaning that they may provide more accurate and precise information about relationships between patient characteristics and outcomes [12,13]. In that regard, while tumor grade is a surrogate marker for clinical outcome, it does not encapsulate the full biologic or clinical diversity of meningioma. Instead, it may be better to predict clinical outcome directly and use grade as an additional feature. To address the need for integrated predictive models for meningioma patients, we used seven ML algorithms to predict clinical outcomes based on preoperative information, conventional prognostic features such as grade and extent of resection, or a combination of both using data from a cohort of 235 patients with 257 meningiomas. In particular, we use standard clinical statistical methods (Logistic and Cox Regression) and recommended ML algorithms for biomedical data where input features are known (CART, MediBoost, Random Forests, Gradient Boosting, and Support Vector Machines) [12,13]. Our results reveal that ML models can estimate the risk of meningioma recurrence and patient survival from information that is available before meningioma resection, but that integrated models incorporating all available demographic, clinical, radiographic and pathologic data provide the best estimates of outcome. From these models, we develop decision trees and nomograms that may be used to individualize treatment for meningioma patients, and provide a framework for using ML analysis for other central nervous system tumors.

## Materials and methods

### Study design and patient population

Patients treated with surgical resection for meningioma at a single institution from 1990 to 2015 were retrospectively identified from a prospective tissue biorepository. Only patients with sufficient tissue for re-grading were included, and all meningiomas were re-evaluated by neuropathologists using contemporary diagnostic criteria [14]. Demographic and clinical information were extracted from the medical record, and patients without either were excluded. These stringent inclusion criteria assured that only cases with thorough and accurate data were included in our analyses. Diagnostic imaging was reviewed for all patients to confirm meningioma location and extent of resection, and perform volumetric analysis with three-dimensional (3D) contours that were manually generated by a single radiation oncologist with expertise in radiotherapy for meningioma (D.R.R.) using MIM Vista version 6.4.9 (MIM Software, Inc., Cleveland, OH). Meningiomas that occupied more than one anatomic location were counted in each location for analysis. With respect to meningioma recurrence after gross total resection, local recurrence of any size was scored on subsequent brain imaging. After subtotal resection, Response Evaluation Criteria In Solid Tumors (RECIST) criteria were adapted to define progression of residual meningioma as interval growth of ≥20% along any dimension. Local failure (LF) and overall survival (OS) were quantified from the date of meningioma resection until the date of tumor recurrence, or death, respectively, or the date of last contact for patients who were alive and without radiographic evidence of recurrence. Survival status of patients was collected by a combined search of the electronic medical record, institutional cancer registry, Surveillance, Epidemiology & End Results Program (SEER), Department of Motor Vehicles (DMV), social security and nationwide hospital obituary databases, as well as a search for publically available obituaries. This study was approved by the Institutional Review Board, Human Research Protection Program Committee on Human Research, and written informed consent for study inclusion was obtained from patients at the time of surgery. Raw, re-identified data are available in S1 Table.

### Features

Three sets of predictors were used to develop models: (1) preoperative clinical features alone; (2) postoperative clinical features, which are conventionally used to stratify meningioma patients; and (3) a combination of both preoperative and conventional postoperative features. Preoperative features included demographic information (age, sex and race), past medical history (prior history of therapeutic radiation to the head or neck, including a prior history of meningioma treatment) and radiographic characteristics derived from computed axial tomography scans and magnetic resonance imaging (meningioma size from 3D volumetric contours, intratumoral necrosis as denoted by low-intensity MRI signal, presence of multiple meningiomas, meningioma invasion of bone or brain, peri-meningioma edema and meningioma location within the anterior cranial fossa, middle cranial fossa, posterior cranial fossa, midline, convexity and/or skull base). Radiographic characteristics, including bone or brain invasion and necrosis, were based on physician assessment of preoperative imaging and are not pathology-verified tissue characteristics. They reflect information that would be commonly available to a physician at that point in time. Models based on conventional postoperative features included patient age, race, sex, meningioma grade, extent of resection and adjuvant radiotherapy.

### Statistical analysis

Kaplan-Meier estimates were plotted to visualize 5-year probabilities of LF and OS. Models were fit on the full cohort and also grouped by grade and extent of resection. Heatmaps were created based on pairwise Pearson correlations and ordered using hierarchical clustering to explore the relationships among features and outcomes of LF or OS.

Multivariate models were trained to predict outcomes of LF or OS. For each outcome, we trained three sets of models based on (1) preoperative features (“preoperative models”), (2) postoperative features (“conventional models”), and (3) combined preoperative and postoperative features (“integrated models”). In each case, models were trained using multiple algorithms for two reasons: (1) to compare their performance as no individual algorithm is guaranteed a priori to perform best across all clinical scenarios [11], and (2) to take advantage of the unique ways different algorithms allow us to derive insights from our data. Throughout this text, we use the word model to refer to the estimated mathematical relationship linking a specific set of features and an outcome. In contrast, we use the word algorithm to refer to the procedure used to build, or train, a model. The following algorithms were used: logistic regression (generalized linear model, GLM), classification and regression trees (CART) [15], logistic regression with elastic net regularization (GLMNET) [16], support vector machines (SVM) with a radial basis kernel [17], MediBoost Tree-Structured Boosting [12], random forest (RF) [18] and gradient boosting machine (GBM) [19]. The following packages were used within the rtemis package: GLM: base R; Elastic net: glmnet; SVM: e1071; CART: rpart; MediBoost: rtemis implementation; Random forest: ranger. We chose these to include the most common modeling tool used in clinical medicine to date, logistic regression, along with ML approaches, each of which are well suited for structured data (in contrast to deep learning, which would be applicable on unstructured data, such as raw medical images). All models were trained and tested using nested resampling to minimize overfitting. For each outcome and feature set combination, 100 stratified subsamples were generated (outer resampling), where the full sample was split into three quarters that were used for training, and one quarter that was left out for testing. The same sets of subsamples were used across algorithms to make their performance comparable. Model tuning was performed by grid search of hyperparameters and 5-fold cross-validation of the training set (inner resampling). In this way, test sets were never seen during model building and only used to estimate model performance. The performance of each model was assessed by calculating the balanced accuracy on each left-out set, which is a measure of classification performance designed for skewed class distributions, and is defined as the mean of sensitivity and specificity of a model [20]. Finally, the mean and standard deviation of the balanced accuracy obtained from each of the 100 subsamples was calculated for each set of models.

Decision Trees were built using MediBoost on the whole sample after tuning on five stratified subsamples. Nomograms were built to estimate 5-year freedom from local failure and overall survival using penalized Cox regression models trained with an adaptive elastic net procedure using 10-fold cross-validation on the whole sample [21]. The adaptive elastic net is an adaptation of the original Cox regression for survival analysis which can handle correlated features and performs variable selection. Nomogram performance was assessed on 100 bootstrap samples. Finally, a random forest model was trained on preoperative clinical features using the full sample and was used to power an online LF risk calculator. Table 1 highlights and summarizes some of the algorithms’ main characteristics as they relate to clinical modeling.

**Table 1:** Algorithms.

Algorithm	Type	Selected characteristics	Interpretability
Logistic regression (generalized linear model, GLM)	Classification	Most commonly used model in medical literature. Models linear relationships, requires uncorrelated features	++++
Logistic regression with elastic net regularization (GLMNET)	Classification	Adaptation of logistic regression to handle correlated features (as well as high-dimensional datasets). Among correlated features, some will be dropped entirely from the model, even if predictive	+++
Support Vector Machine—SVM	Classification	Popular ML tool in biomedical research offers competitive performance among multiple datasets but poor interpretability	+
Classification and Regression Trees—CART	Classification	Builds an intuitive decision tree for easy patient stratification. Automatically models feature interactions	++++
Tree-Structured Boosting—MediBoost	Classification	Same structure as CART (builds a single decision tree), but with improved accuracy by considering weighted versions of all cases at each split	++++
Random Forest (RF)	Classification	Best out-of-the-box performance with no tuning. Variable importance suggests features that contribute to prediction after considering interactions, but no directionality or explicit interactions shown	++
Gradient Boosting Machine (GBM)	Classification	Best overall performance on structured data across real-world applications. Variable importance similar to RF	++
Penalized Cox regression (Adaptive Elastic Net)	Survival Analysis	Allows Cox survival analysis with high dimensional, correlated data and building of clinically-interpretable nomograms. As in classification, among highly correlated features, some may be dropped from the model, even if predictive.	+++

All predictive modeling and visualization was performed using the rtemis package for machine learning and visualization (https://egenn.github.io/rtemis) in R (The R Project for Statistical Computing, https://www.r-project.org). Nomograms were created using the hdnom package [21].

## Results

### Patients, meningiomas, treatments and outcomes

**Fig 1:**
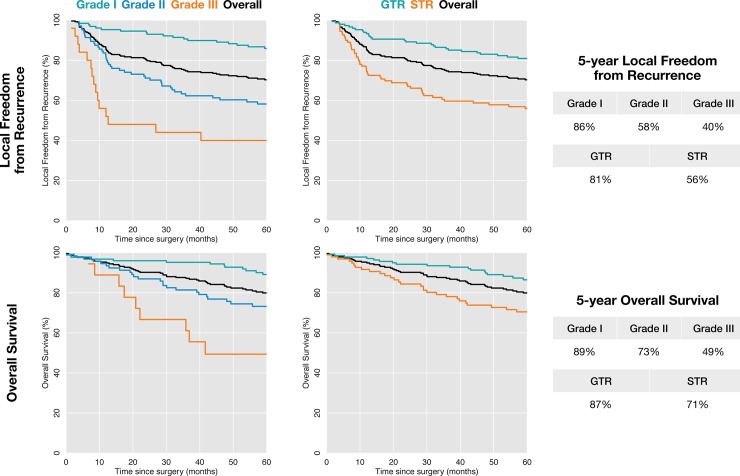
Meningioma outcomes according to conventional predictive factors. Kaplan-Meier estimates for local freedom from recurrence (top) and overall survival (bottom) following surgery for meningioma according to conventional predictive factors of meningioma grade (left) and extent of resection (right).

We identified 235 patients with 257 surgically treated meningiomas with available clinical follow-up data and tissue for re-grading that were eligible for this study (Table 2). The median age at presentation was 59 years (range: 14–87 years). One-hundred fifty patients were female (63.8%) and 85 were male (36.1%). Sixty-eight meningiomas (26.5%) were recurrent. One-hundred fifty meningiomas had evidence of radiographic edema (58.3%), 88 had evidence of radiographic bone invasion (34.2%) and 47 had evidence of brain invasion (18.2%). The median meningioma size was 33.4 cm^3^ (range: 0.30–335 cm^3^), as calculated from three-dimensional volumetric contours. GTR was achieved in 147 cases (57.2%), and 61 meningiomas received adjuvant radiation (23.7%). There were 128 WHO grade I (49.8%), 104 WHO grade II (40.4%) and 25 WHO grade III meningiomas (9.7%). With a median follow-up of 4.3 years (range: 0–16 years), the Kaplan-Meier 5-year local freedom from recurrence estimates were 86%, 58% and 40%, and the 5-year overall survival estimates were 89%, 73% and 49% for WHO grade I, WHO grade II and WHO grade III meningiomas, respectively (Fig 1).

**Table 2:** Patients, meningiomas, treatments and outcomes.

Patients	235
Median age (range)	58.6 years (13.7–86.5 years)
Male:Female (ratio)	85:150 (1:1.8)
History of head or neck radiotherapy	11 (5%)
Multiple meningiomas	54 (23%)
**Race**	
Caucasian	157 (67%)
Hispanic	25 (11%)
Asian	22 (9%)
Black	12 (5%)
Other (not Hispanic/Latino)	6 (3%)
Pacific islander	5 (2%)
White; Hispanic/Latino	2 (1%)
**Meningiomas**	257
World Health Organization (WHO) grade I	128 (50%)
WHO grade II (atypical)	104 (40%)
WHO grade III (anaplastic)	25 (10%)
Primary:recurrent (ratio)	189:68 (2.8:1)
Median size (range)	33.4 cm^3^ (0.3–335.3 cm^3^)
Bone invasion	88 (34%)
Brain invasion	47 (18%)
Peri-meningioma edema	150 (58%)
**Location**	
Anterior cranial fossa	54 (21%)
Middle cranial fossa	58 (23%)
Posterior cranial fossa	34 (13%)
Midline	118 (46%)
Convexity	157 (61%)
Skull base	109 (42%)
**Treatment**	
Extent of resection	
Gross total resection	147 (57%)
Subtotal resection	109 (42%)
Extent of resection unknown	1 (0.4%)
Adjuvant radiotherapy	61 (24%)
**Outcomes**	
Median follow-up (range)	52 months (0–197 months)
Local failure	92 (36%)
Median local freedom from progression (range)	76 months (1.7–207 months)
Death	60 (27%)
Median overall survival (range)	80 months (0–191 months)

### Hierarchical clustering of meningioma features reveals correlations with clinical outcomes

**Fig 2:**
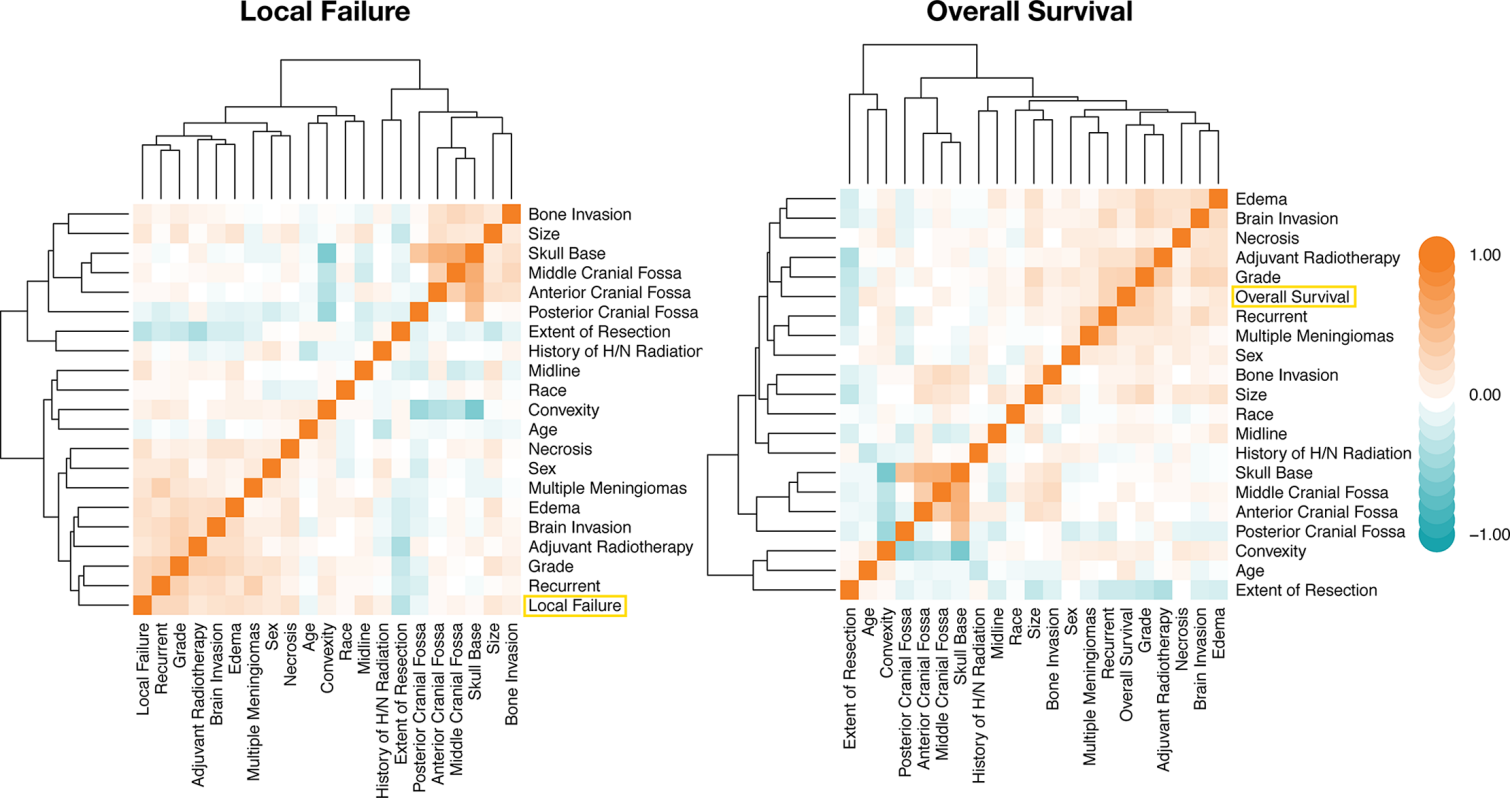
Meningioma feature correlation heatmaps. Heatmaps based on features’ pairwise Pearson correlation. Rows and columns have been arranged by hierarchical clustering to reveal each feature’s relationship to the outcome of interest. Orange denotes positive correlations, and teal indicates negative correlations.

To explore the relationships among demographic, clinical and radiographic features and meningioma outcomes, we constructed heatmaps based on pairwise Pearson correlations of preoperative and postoperative features (Fig 2). As expected, LF was most closely related to meningioma grade (r = 0.31) and setting (primary versus recurrent; r = 0.31), which were highly correlated with one another as well (r = 0.36). LF was also correlated with peri-meningioma edema (r = 0.24), intra-meningioma necrosis (r = 0.22) and brain invasion (r = 0.21). Similarly, overall survival was most closely related to meningioma grade (r = 0.34) and setting (r = 0.31), but was also highly correlated with remote history of adjuvant radiotherapy (r = 0.21) and increasing meningioma size (r = 0.20). In sum, our quantitative analysis of correlations among meningioma features corroborates the qualitative clinical impressions held amongst physicians regarding meningioma outcomes in terms features such as recurrent meningioma and meningioma size, among others, correlating with worse LF [22]. Thus, the dataset assembled for this study is representative of the larger population of meningioma patients seen in tertiary care, and is suitable for ML analysis.

### Preoperative features predict meningioma outcomes

**Fig 3:**
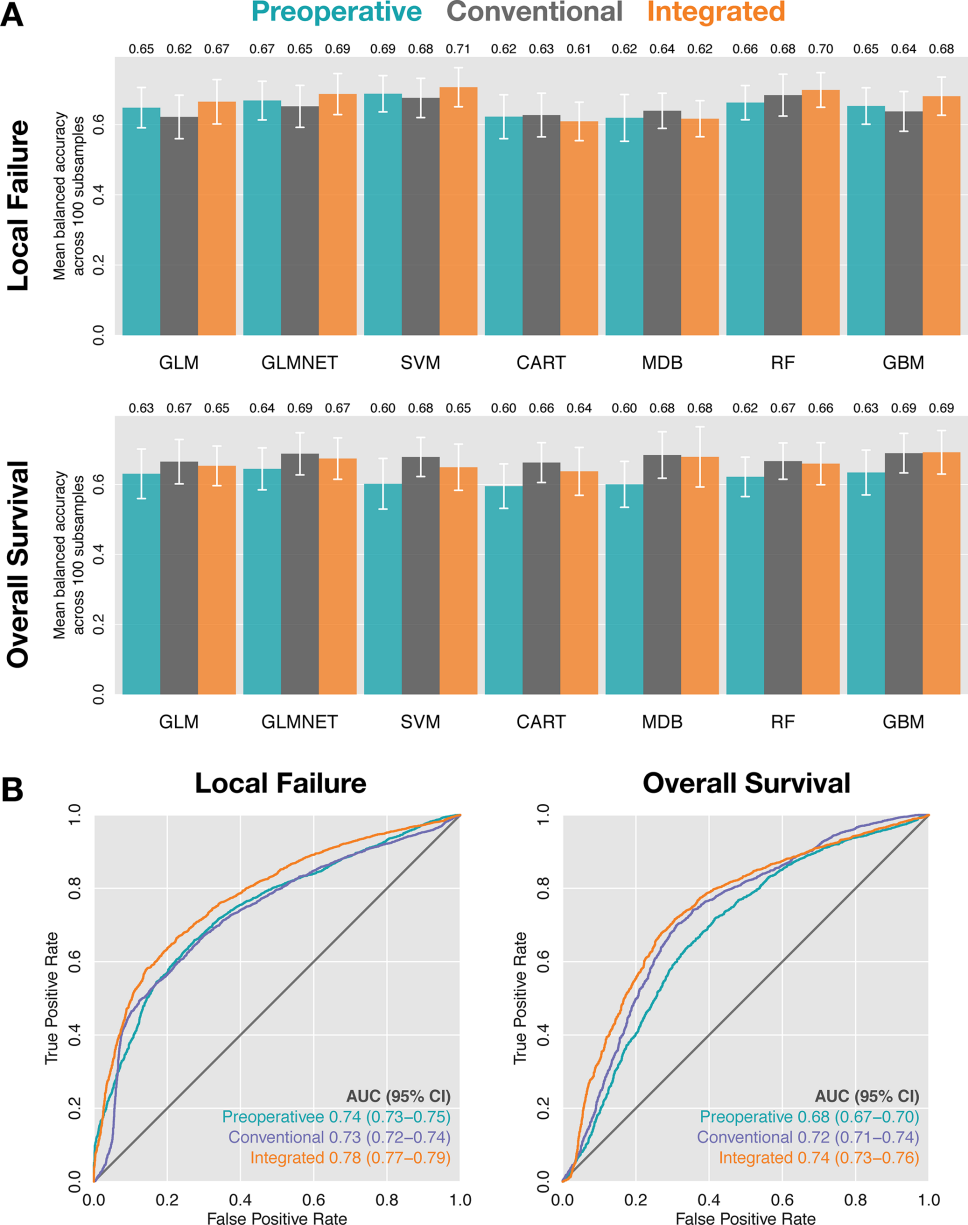
Machine learning model accuracy. (A) Mean balanced accuracy across 100 subsamples of models predicting local failure (top) and overall survival (bottom). Error bars indicate standard deviation. (B) Receiver-operator characteristic curves for local failure (left) and overall survival (right) for the best preoperative (SVM and GLMNET, respectively), conventional (RF and GLMNET, respectively) and integrated models (SVM and GBM, respectively) as defined in (A). 95% confidence intervals were estimated using 2000 bootstraps. All results are reported on left-out test sets not used for model training.

A comprehensive ML analysis was applied to predict meningioma outcomes based on preoperative data, conventional prognostic features (patient age, race and sex; and meningioma grade, extent of resection and adjuvant radiotherapy), or a combination of both preoperative and conventional data. All models were trained and tested by nested resampling, using seven algorithms: logistic regression (generalized linear model, GLM), logistic regression with elastic net regularization (GLMNET), support vector machines (SVM) with a radial basis function, classification and regression trees (CART), MediBoost Tree-Structured Boosting, random forest (RF) and gradient boosting (gradient boosting machine, GBM). Area under the ROC curve was estimated for each model and 95% confidence intervals were estimated using 2000 bootstraps. On average, the top models based on preoperative data slightly outperformed the top models based on conventional prognostic features in predicting LF (Fig 3A). In contrast, conventional models outperformed preoperative models in the prediction of OS. In both cases, integrated models were the top performers. Specifically, the top conventional model (RF) predicted LF with an average balanced accuracy of 0.68 (AUC = 0.73; 95%CI = 0.72–0.74), compared to the top preoperative model (SVM) with an average balanced accuracy of 0.69 (AUC = 0.74; 95% CI = 0.73–0.75) and the top integrated model (SVM) with an average balanced accuracy of 0.71 (AUC = 0.78; 95% CI = 0.77–0.79) (Fig 3B). In the prediction of OS, the top conventional model achieved an average balanced accuracy of 0.69 (AUC = 0.72; 95% CI = 0.71–0.74), the top preoperative model achieved an average balanced accuracy of 0.64 (AUC = 0.68; 95% CI = 0.67–0.70), and the integrated model achieved an average balanced accuracy of 0.69 (AUC = 0.74; 95% CI = 0.73–0.76) (Fig 3B). These results indicate that the risk of meningioma recurrence, and to a lesser extent, overall survival, can be estimated using information that is available before a patient is ever taken to the operating room.

### Variable importance, decision trees and nomograms illustrate the clinical utility of machine learning algorithms for individualized meningioma treatment

**Fig 4:**
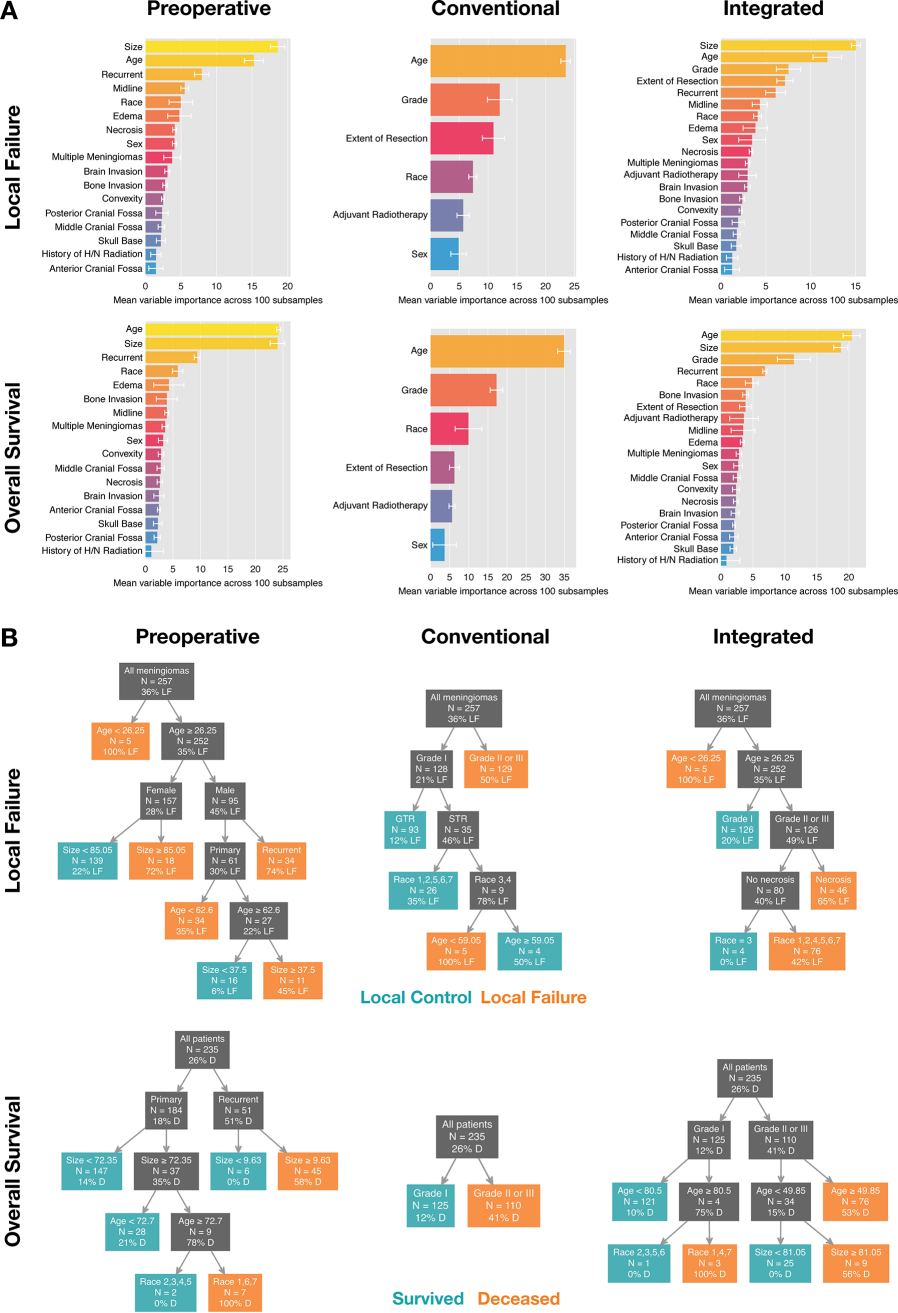
Variable importance and decision trees. (A) Mean variable importance derived from 100 random forest models predicting local failure (top) and overall survival (bottom) for preoperative (left), conventional (middle) and integrated (right) models. Error bars indicate standard deviation. (B) Decision trees built using MediBoost Tree-Structured Boosting predicting local failure (top) and overall survival (bottom) corresponding to preoperative (left), conventional (middle) and integrated models (right). N indicates number of meningiomas (LF) and number of patients (OS) that fall into each branch and percentage indicates proportion of those with local failure or deceased, respectively. Unlike tradition decision trees, MediBoost chooses splits based on weighted versions of the full sample at each node, making splits more reliable even as tree depth increases. PCF: posterior cranial fossa; 1, Caucasian; 2, Black; 3, Asian; 4, Hispanic; 5, Pacific Islander; 6, Other; not Hispanic/Latino; 7, White; Hispanic/Latino.

Random forest (RF) offers built-in estimation of each feature’s variable importance [21]. This is an estimate of each variable’s contribution to the final prediction after considering potential high-level interactions. Mean variable importance of preoperative, conventional and integrated meningioma features in predicting LF and OS after averaging across our 100 subsamples is shown in Fig 4A. To facilitate rapid visual stratification of meningioma patients in a clinical setting according to ML models, we explored decision tree-based algorithms to predict LF and OS. Random forest (RF) was trained with 500 trees, and gradient boosting (GBM) resulted in more than 5,000 trees after tuning, neither of which can be explicitly interpreted. Drawbacks of RF- and GBM-derived variable importance scores include that they do not suggest directionality and do not reveal the nature of variable interactions. These are both addressed by single decision trees. CART and MediBoost both build a single tree, which makes each of these models highly interpretable. Between them, MediBoost had the highest balanced accuracy and even surpassed random forest in 2 out of 6 cases: conventional and integrated models of OS (Fig 4B). MediBoost uses a procedure where, in contrast to CART and similarly to GBM, weighted versions of all cases are used to derive splits at each point in the tree, which can be advantageous particularly in relatively small datasets like the current one. In that regard, MediBoost is superior to traditional decision trees estimated using recursive partitioning insofar as the latter suffer from exponential decrease of available data at each level.

**Fig 5:**
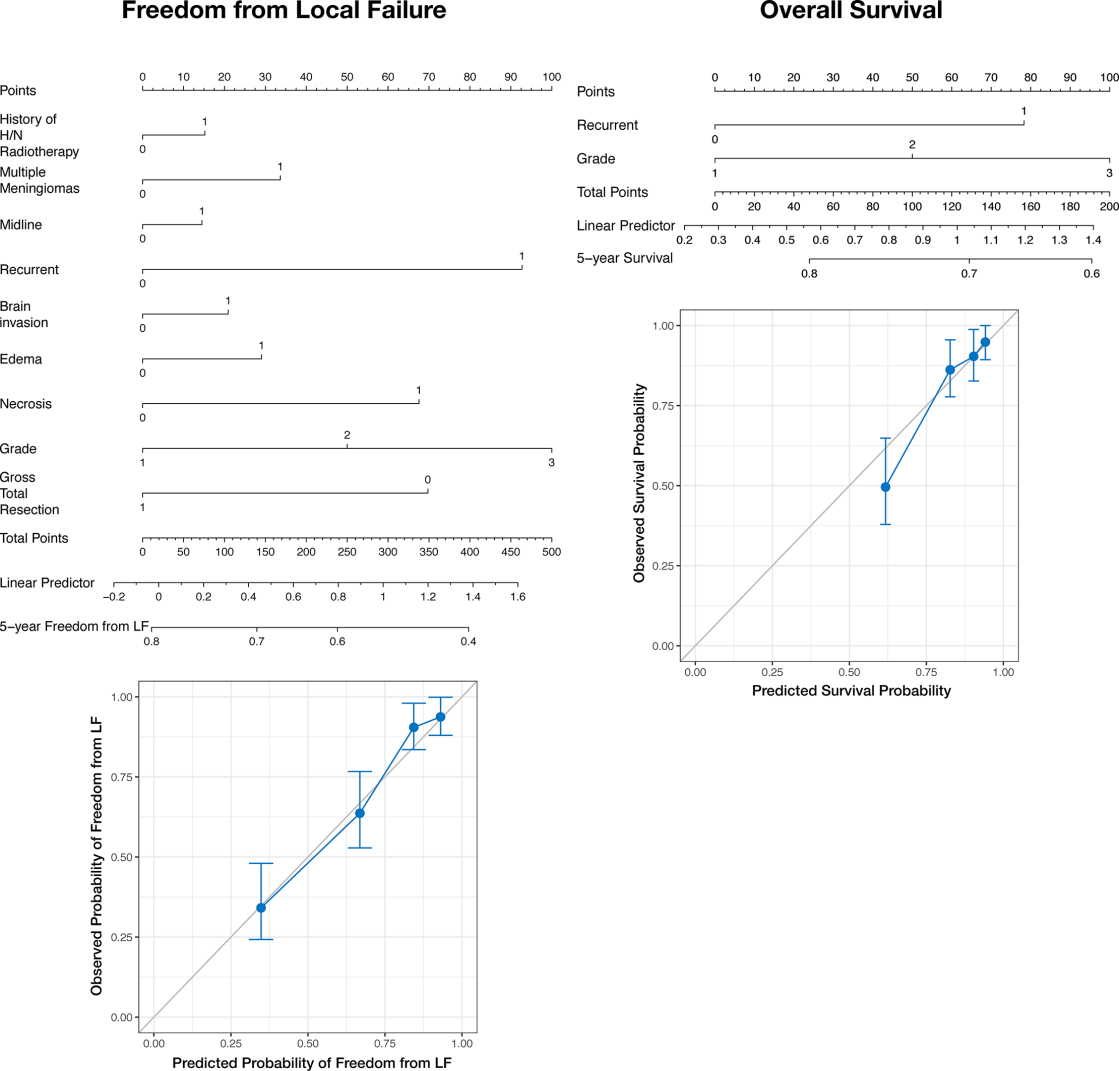
Machine learning nomograms for meningioma outcomes. Nomograms built using a penalized Cox model (adaptive elastic net) to predict 5-year freedom from LF (left) and 5-year survival (right) on the full sample. This procedure provides accurate survival estimates even in the presence of correlated features, which are not allowed in the original Cox regression model. Scatter plots show observed versus predicted probabilities obtained by training on 100 bootstrap samples and testing on the left-out set.

Additionally, we used the adaptive elastic net trained with 10-fold cross validation on integrated preoperative and conventional feature sets to perform multivariate survival analysis of LF and OS. These models were used to construct a pair of nomograms, which were internally validated on 100 bootstrap resamples (Fig 5). Finally, an online interactive risk calculator for LF was created based on a random forest model trained on the full sample of preoperative data and can be accessed at https://egenn.shinyapps.io/Meningioma_LF_Risk_Calculator/.

## Discussion

Meningioma outcomes are influenced by myriad patient, tumor and treatment-specific factors, but clinical decisions regarding meningioma patients are often dominated by tumor grade and extent of resection [22]. Here, we perform a comprehensive ML analysis using multiple algorithms (commonly used statistical methods and modern ML algorithms), each with their respective advantages (Table 1), to predict meningioma outcomes of LF and OS from demographic, clinical, radiographic and pathologic data. To do so, we developed an integrated database containing information from 235 patients who underwent surgery for 257 meningiomas at a single institution over a 25-year period. Our results reveal that models restricted to preoperative information, such as patient demographics and radiographic features, have similar accuracy for predicting LF or OS as models based on meningioma grade and extent of resection. RF models of LF and OS produced a similar ranking of feature importance, with larger meningioma size and greater patient age (continuous variables) occupying the top two positions in both preoperative and integrated models. Consistent with prior investigations, meningioma setting (primary or recurrent) and meningioma grade had high variable importance in preoperative and integrated models, respectively [22]. Conventional models featured age and grade as the dominant features in predicting both LF and OS. Our variable importance analysis of predictive models for meningioma outcomes corroborates clinical and empiric experience with meningioma treatment, further supporting the suitability of ML as a valuable adjunct to clinical decision making.

Integrating demographic, clinical, radiographic and pathologic data, we develop easy to use decision trees and nomograms to readily identify patients with different underlying risks of LF or OS. These tools may serve as a guide to select meningioma patients who are most likely to benefit from close clinical surveillance versus adjuvant treatment after resection, and may also provide a framework for ML analysis of other central nervous system tumors. With respect to the former, delaying or omitting adjuvant radiotherapy obviates the risks of neurocognitive decline and secondary malignancies, and the models we report may minimize overtreatment of meningioma patients. In sum, our predictive models can be used as a decision support tool in combination with clinical experience and patient preference to determine the best management strategy for each individual. Nevertheless, additional analyses with a higher number of patients and multiple institution validation should be performed to increase the reliability of our results.

Clinical research studies commonly depend on a single algorithm for data analysis [10]. In the present study, we used seven algorithms, which provided a better estimate of the performance of our three sets of models (preoperative, conventional and integrated) in predicting LF or OS than any single algorithm. Moreover, we were able to extract clinically significant information from our data by capitalizing on the strengths of each algorithm used. In that regard, we used random forests to investigate variable importance, and MediBoost to plot decision trees for all feature sets and outcomes. Each of these analyses were facilitated by the robust database containing a relatively large number of patients and features with both internal and external validity that was developed for this study. Importantly, all of the meningiomas included in this study were re-evaluated according current diagnostic criteria, which increases the generalizability of our results [3]. Indeed, the lack of large meningioma databases organized by contemporary World Health Organization standards has been a major barrier to meningioma research [22], but we were able to overcome this obstacle through multidisciplinary collaboration within our institution which included histopathologic re-review of all the cases included in this work.

A main limitation of this study is that our database was retrospectively assembled from patients treated at a single institution. Thus, our results and conclusions should be interpreted with potential selection and information biases in mind. It should also be noted that the accuracy of the predictive models we develop are not perfect, and would be improved by increasing the number of patients in the study and the addition of radiomic or biologic features, as has been demonstrated by other investigators for prediction of meningioma grade [10,23]. Indeed, the magnetic resonance characteristics of meningiomas have high sensitivity and specificity for meningioma grade and histopathologic subtype [24,25]. Crucially, training and testing of ML algorithms on larger and more diverse data sets would allow better model estimation, with less institutional bias and better estimates of model generalizability. It should be noted that, as is common in clinical modeling, race is likely serving as a proxy to multiple demographic, genetic, and environmental features not present in our database and should be cautiously interpreted as such. Our nomograms were trained on the full sample to produce a single model, and although 10-fold cross-validation was used for training and 100 bootstraps were used to test its stability, proper validation requires an external dataset. The aforementioned barriers to accurate meningioma diagnosis across different histopathologic eras have precluded our efforts to assemble larger data sets thus far, but we are hopeful that modern prospective meningioma trials will yield valuable and accurate data sets for future predictive model refinement [8,9].

## Supporting information

S1 Table Raw, re-identified data used in this study. (CSV)
